# Citric Acid Induces the Increase in Lenthionine Content in Shiitake Mushroom, *Lentinula edodes*

**DOI:** 10.3390/foods11244110

**Published:** 2022-12-19

**Authors:** Mengting Hong, Dan Han, Jinjin Qiao, Xiaolin Zhou, Hanshou Yu, Liang Shi

**Affiliations:** Key Laboratory of Microbiological Engineering of Agricultural Environment, Ministry of Agriculture, Department of Microbiology, College of Life Sciences, Nanjing Agricultural University, Nanjing 210095, China

**Keywords:** *Lentinula edodes*, lenthionine, citric acid

## Abstract

Shiitake mushroom, *Lentinula edodes*, is the second largest edible fungus in the world, with a characteristic aroma. 1,2,3,5,6-pentathioheterocycloheptane, commonly known as lenthionine, is the main source of this aroma. Lenthionine has high commercial value, and if we explore the possible induction mechanism of citric acid in lenthionine synthesis, we can provide a reference for the effective application of citric acid as an inducer. In this paper, the single-factor treatment of *Lentinula edodes* with variable citric acid concentration and treatment duration showed that the best citric acid concentration for *L. edodes* was 300 μM, and the best treatment duration was 15 days. Additionally, the optimal design conditions were obtained using the response surface method (RSM); the treatment concentration was 406 μM/L, the treatment duration was 15.6 days, and the lenthionine content was 130 μg/g. γ-Glutamyl transpeptidase (LEGGT) and cystine sulfoxide lyase (LECSL) are the key enzymes involved in the biosynthesis of lanthionine. The expression levels of LEGGT and LECSL genes increased significantly under citric acid treatment. Additionally, the lenthionine content of the silenced strains of LEGGT and LECSL was significantly decreased.

## 1. Introduction

*Lentinula edodes* originated in China and is an edible fungus species widely grown across Asia. Research on the volatile compounds in *L. edodes* shows that 25 volatile sulfur-containing compounds (VSC), 8 carbon-containing compounds, and aldehydes contribute to the flavor of *L. edodes* [1]. Among them, 1,2,3,5,6-pentathiepane is considered to be the most important substance in *L. edodes*, due to its unique odor. This pentasulfide heterocyclic compound is commonly known as “lenthionine” [2]. The preference for this unique aroma greatly affects the choice of *L. edodes* by consumers in different regions [3,4]. The results of Morita et al. [5] show that lenthionine existed stably before 225 °C and could be used safely as a spice in cooking. Lenthionine is a kind of all-natural and healthy condiment, which can encourage profit growth points for food enterprises. 1,2,3,5,6-pentathiaheptane is a standard for evaluating the quality of *Lentinula edodes*, but its low content and the difficulty of its industrial synthesis limit its commercialization. Existing research on improving the content of *Lentinula edodes* essence mainly focuses on two aspects, one being post-harvest treatment, and the other being the improvement of biosynthesis. In terms of post-harvest treatment, a study by Zhang et al. [6] used freeze drying, hot-air drying, and natural drying to dehydrate fresh *L. edodes*, and the drying process changed the volatile compound content. The influence of post-harvest treatment on the content of lenthionine is not stable, and it is very easy to cause the volatilization of lanthionine [7]. Therefore, the improvement in the level of biosynthesis is considered to be the key to increasing the content of lenthionine. It is of great significance to improve the level of lenthionine biosynthesis and analyze the synthesis mechanism, in order to improve its commercial value.

**Figure 1:**
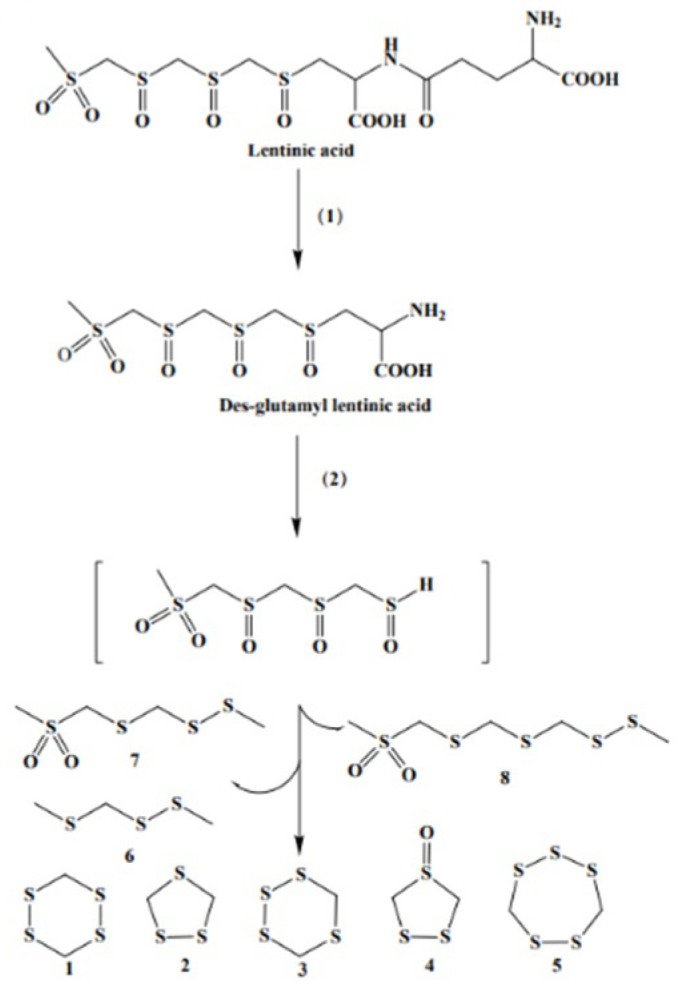
Pathway for the formation of sulfurous flavor compounds from lentinic acid in *L. edodes*. The lenthionine is produced from the starting substrate lentinic acid following a two-step catalytic process of (**1**) and (**2**) and through a complex non-enzymatic reaction. **1** 1,2,3,5-tetrathiane; **2** 1,2,4-trithiolane; **3** 1,2,4,5-tetrathiane; **4** 1,2,4-trithiolane-4-oxide; **5** lenthionine; **6** 2,3,5-trithiahexane; and **7** 2,3,5,7-tetrathiaoctane-7,7-dioxide; 8 2,3,5,7,9-pen-tathiadecane-9,9-dioxide. (**1**) γ-glutamyl transpeptidase and (**2**) cysteine-sulfoxide lyases.

Lenthionine was first reported by Morita in 1966 as a volatile cyclic sulfur compound, with the molecular structure formula of C_2_H_4_S_5_ [5]. Yasumoto et al. [8] first proposed a possible metabolic pathway of lenthionine, and believed that lenthionine was produced by lentinic acid through a two-step enzyme-catalyzed reaction and various non-enzymatic reactions. Yasumoto et al. [9] improved the metabolic pathway of lenthionine, and identified the enzymes and metabolites involved in the reaction pathway. The key enzyme γ-glutamyl transpeptidase (LEGGT) hydrolyzes the glutamine peptide bond in lentinic acid to produce glutamyl lentinic acid, and glutamic acid as a by-product. Cystine sulfoxide lyase (LECSL) acts to further convert the product into thiosulfinate, which is an unstable and highly reactive intermediate. A non-enzymatic reaction occurs at the end of the synthesis route, which further degrades to produce the intermediate of dithioheterocyclic propane, formaldehyde, and other by-products. The intermediate of dithioheterocyclic propane is then polymerized into hexathiol heterocyclic heptane, tetrathioheterocyclic heptane, lanthionine, and other products. The synthesis mechanism is shown in Figure 1. However, the regulatory mechanism of the two key enzymes in the biosynthesis of lenthionine remains unclear. Iwami et al. [10] showed that glutamyl transferase was involved in the synthesis mechanism of lenthionine from lentinic acid. The transferase produced desglutamyl lentinic acid, either by transfer or by the hydrolytic reaction. However, it is not clear how the glutamyl moiety of the substrate is favorably hydrolyzed or is transferred to the acceptor, lentinic acid. One of the most promising candidates for this function is the α-amino group on the enzyme, and further studies are needed to determine the mechanism of the enzyme’s action and to identify the particular group functioning as the acceptor site for the γ-glutamyl moiety of the substrate.

The term ‘exogenous inducers’ mainly refers to the biological factors that can stimulate microorganisms to produce secondary metabolites to resist the stress response. The target of the inducer may be a gene, or an active group of an enzyme, etc. Therefore, changes in energy metabolism or reaction efficiency result in the accumulation or inhibition of secondary products. The most commonly used induction factors are salicylic acid, chitosan, various organic acids and inorganic salts, etc. [11]. Cao et al. [12] showed that the addition of 200 µM of salicylic acid significantly increased the content of ganoderic acid, an important secondary metabolite in *Ganoderma lucidum*. Ren et al. [13] found that treatment with 5 mM of acetic acid for 72 h increased the content of ganoderic acid. Small-molecule organic acids can improve the level of biosynthesis in terms of increasing the level of the induced substrate and the activity of various enzymes in the induced biosynthesis pathway. Zhang et al. [14] found that adding an appropriate amount of acetic acid, pyruvic acid, fumaric acid, and citric acid to the culture medium can promote the synthesis of 2,3-butanediol in *Serratia marcescens* to a certain extent, and acetic acid as an inducer could increase the activity of B-galactosidase by nearly four times. Citric acid (CA) is a tricarboxylic acid (TCA) compound. Intracellular citric acid is involved in an important metabolic pathway, the tricarboxylic acid cycle, and its derived TCA cycle intermediates are closely involved in a complex metabolic network consisting of biohormones, amino acids, and sugar biosynthesis [15]. The results of Liu [16] show that the exogenous addition of citric acid induced the production of secondary metabolite intracellular polysaccharides in *Agaricus bisporus*. Whether exogenous citric acid affects the content of lenthionine, a secondary metabolite of *L. edodes*, is less studied and needs to be further explored.

The market demand for *L. edodes*, as an edible fungus with a rich aroma and high nutritional value, is increasing year by year. The *L. edodes* industry needs more high-quality products while increasing its output. Lenthionine is an all-natural and healthy condiment. With the constant pursuit of people’s health, lenthionine will promote the development of food enterprises. The commercial value of lenthionine as a flavoring agent is high, but its commercial development is limited. A current development trend in the industry is to promote the research of lethenionine. There are few studies on citric acid as an inducer to regulate fungal growth and development, and secondary metabolites, especially in *L. edodes*. If we further explore the possible induction mechanism of citric acid in lenthionine synthesis, we can provide a reference for the effective application of citric acid as an inducer in fungi. If the low-cost application of a citric acid inducer can effectively improve the quality and cultivation efficiency of *Lentinula edodes*, this will result in great progress in the development of the *Lentinula edodes* industry.

## 2. Materials and Methods

### 2.1. Strain and Material Culture

*L. edodes* (NACC6250, from the Jiangsu Agricultural Microbial Germplasm Resources Collection of China) was used as the wild-type (WT) strain. The WT, CK (empty-vector controls), and LEGGT-silenced, LECSL-silenced *L. edodes* strains were cultured at 25 °C in a solid complete yeast medium (CYM). The formulation of the CYM medium was based on the method of Zhang et al. [17]: 1% maltose (biochemical grade, Sangon, Shanghai, China), 2% glucose (biotech. grade, Sangon, Shanghai, China), 0.2% yeast extract (for microbiological grade, Sangon, Shanghai, China), 0.2% peptone (for microbiological grade, Sangon, Shanghai, China), 0.05% MgSO_4_·7H_2_O (biochemical grade, Sangon, Shanghai, China), and K_2_HPO_4_ (biochemical grade, Sangon, Shanghai, China).

After culturing for 10 days in a constant-temperature incubator at 25 °C, ten pieces of mycelial blocks were aseptically picked and used to inoculate 100 mL of CYM, then incubated in an oscillator at 25 °C and 100 rpm/min for 7 days. Subsequently, the mycelial blocks were evenly broken in aseptic conditions, and 10 mL of broken mycelium was pipetted into 100 mL of CYM liquid medium. The culture was continued for 25 days in an oscillator at 25 °C and 100 rpm/min.

### 2.2. Citric Acid Treatment

Citric acid was dissolved in deionized water, and the concentrations of citric acid used were 100 µmol/mL, 200 µmol/mL, 300 µmol/mL, 400 µmol/mL, 500 µmol/mL, and 600 µmol/mL. The same volume of solution with different concentrations was added to the CYM medium, according to the concentration gradient of the inducer, and each treatment was repeated three times, with deionized water used as the control. The fermented seed solution was crushed with a crusher and then added to the CYM medium with an inoculum of 10 mL per bottle, before being placed in a shaker at 25 °C.

### 2.3. Determination of Mycelium Biomass

The mycelium was collected and dried in an oven at 40 °C. The weight of the dried mycelium was the mycelial biomass.

### 2.4. Analysis of the Activities of LEGGT and LECSL

The sample was ground in liquid nitrogen, added to 5 mL of Tris-HCl (50 mM, pH = 7.0) buffer, homogenized in an ice bath, and centrifuged at 8000× *g* at 4 °C for 20 min. The supernatant was the crude enzyme solution. The crude enzyme solution was used to assay the enzymatic activities of LEGGT and LECSL, and the method was performed as described by Kimikazu and Li [18,19].

### 2.5. Construction of RNAi Plasmids and Strains

The construction of the RNAi vector and the empty-vector control (CK), the transformation of the *L. edodes* strain, and the random screening of transformants were improved with reference to a previous protocol for *Ganoderma lucidum*, which is described as follows [17]. The fungal RNAi vector, pAN7-dual, carries the hygromycin B (Hyg) resistance gene as a selectable marker [20]. The dual-promoter-silencing vector, pAN7-dual, which is driven by the glyceraldehyde-3-phosphate dehydrogenase (gpd) promoter and the 35S promoter, was used to suppress the expression of LEGGT or LECSL. The LEGGT or LECSL fragment was doubly digested with KpnI and SpeI, and inserted into the plasmid pAN7-dual after KpnI/SpeI restriction digestion. This plasmid was used to transform the protoplast of *L. edodes* via lipofectamine transfection, and then transformants were selected randomly on the CYM medium containing 6 μg/mL Hyg [21]. After the detection of the silencing efficiency, six strains were selected and named leggti-7, leggti-19, leggti-31, lecsli-18, lecsli-24, and lecsli-28.

### 2.6. Real-time PCR Analysis of Gene Expression

The total RNA was extracted from a 10-day-old mycelium using an RNA isolation kit (TaKaRa, Dalian, China) according to the manufacturer’s instructions. The levels of LEGGT-specific mRNA expressed by the WT, CK, and leggti strains, and the levels of *LECSL*-specific mRNA expressed by the WT, CK, and lecsli strains were assessed via qRT-PCR, as previously described [22]. The β-actin gene was used to normalize the expression of the experimental genes [23]. The primers used are listed in the Supplementary Materials, Table S1.

### 2.7. Detection of Lenthionine Content

A previously described method was used for the extraction and purification of lenthionine from dried *L. edodes* and the measurement of its level [24].

**Figure 2:**
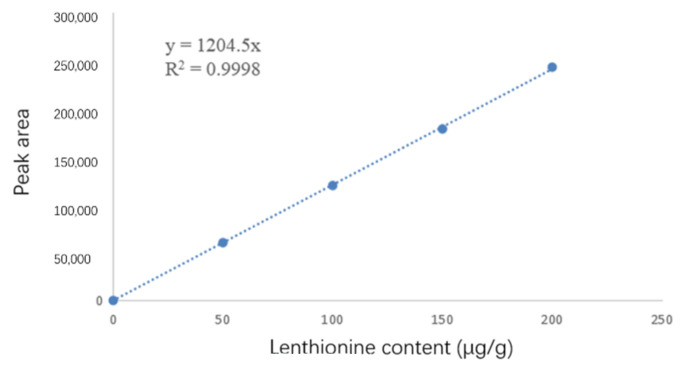
The standard curve of lenthionine.

Dried mycelia (0.1 g) were weighed (drying conditions were 40 °C for 5 h) and ground. One milliliter of methanol solution was added to the sample and centrifuged to obtain the supernatant. Then, the solution was used for the analysis of the lenthionine content. A Shimadzu i-Series high-performance liquid chromatography (HPLC) system, equipped with a diode array detector and a C18 Rapid Resolution HT 18 μm column (2.1 × 50 mm), was used to measure the lenthionine content. Lenthionine was measured using a mobile phase of 50% methanol and 50% ultrapure water at a constant flow rate of 1 mL/min and was monitored at a wavelength of 210 nm. Lenthionine (≥95%, Chembest) was taken as the external standard. The following concentrations of lenthionine were used: 0, 50, 100, 150, and 200 µg/g. The calibration curve was calculated according to the peak area and the corresponding concentration of lenthionine. The standard curve is shown in Figure 2.

### 2.8. Experimental Parameters of Response Surface Method

The single-factor test of citric acid showed that the concentration of citric acid and the length of the treatment duration had an influence on the essence of *L. edodes*. Based on the concentration and time, the response surface method was used to optimize the conditions of citric acid or salicylic acid treatment. There were two test factors in this test, namely concentration and time, so a central composite design (CCD) was adopted. The lenthionine content was taken as the dependent variable (Y), while the treatment duration and concentration of citric acid or salicylic acid were taken as the independent variables (X).

In this experiment, Design-Expert 12.0 software was used to analyze the test data, and mathematical models were used to fit the results [25]:

$$Y=\beta_{k0}+\sum_{i=1}^{5}\beta_{\mathrm{ki}}X_{i}+\sum_{i=1}^{5}\beta_{\mathrm{kii}}X_{i}^{2}+\sum_{i=1}^{4}\sum_{j=i+1}^{5}\beta_{\mathrm{kij}}X_{i}X_{j}$$

*Y*—lenthionine content;
*β*_k0_—intercept;
*β*_ki_—linear effect coefficient;
*β*_kii_—secondary effect coefficient;
*β*_kij_—coefficient of interaction.

The following is the coding equation:

$$X_{i}=\frac{U_{i}-U_{i}^{0}}{\Delta U_{i}}$$

X_i_—coded variable;
U_i_—actual value of variable;
U_i_^0^—actual value of variable center point;
△Ui—gradient of variable.

### 2.9. Statistical Analysis

Each statistical experiment was repeated at least 3 times independently. The experimental data were analyzed using IBM SPSS Statistics 25 (IBM, New York, NY, USA) and plotted with GraphPad Prism 8. Each experimental datapoint shown in the graphs is presented as mean ± SD. The different English letters in the graphs indicate the significant differences between the different treatments; *p* < 0.05 is considered to be significant.

## 3. Results

### 3.1. Effect of Different Concentrations of Citric Acid on Lenthionine and Biomass

**Figure 3:**
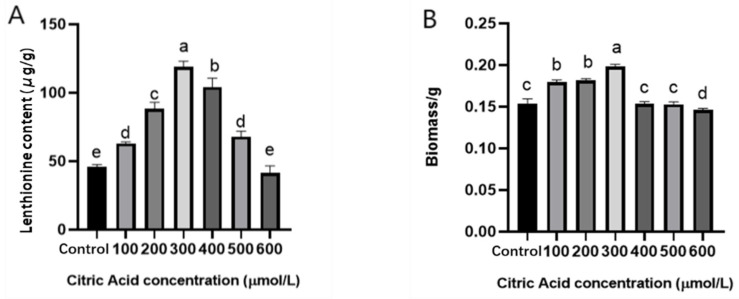
Effects of different concentrations of citric acid on WT lenthionine content and biomass. (**A**) Effects of different concentrations of citric acid on lenthionine content. (**B**) Effects of different concentrations of citric acid on biomass. The calculation results are presented as mean ± standard deviation, and different letters indicate significant differences between different treatments (*p* < 0.05).

In order to investigate the effect of different concentrations of citric acid on the growth and lenthionine of *L. edodes*, different concentrations of citric acid were used as exogenous inducers to treat *L. edodes* mycelium. Lenthionine content, two key enzymes (LEGGT and LECSL), and mycelial biomass were examined at the end of the culture. The lenthionine content was significantly increased by 2.5 times to a value of 118 μg/g when the citric acid concentration was 300 μM, compared with the control group (Figure 3A). The above results indicate that the lenthionine content was significantly induced by citric acid and the effect of citric acid treatment on *L. edodes* showed a concentration effect. The highest biomass of *L. edodes* was 0.2 g at 300 μM citric acid, which increased the biomass compared with the control group, indicating that citric acid not only elevated the content of lenthionine, but also had some growth-promoting effects on *L. edodes* (Figure 3B). This suggests the possibility of citric acid as an inducer for practical applications in production.

### 3.2. Effect of Citric Acid on Lenthionine and Biomass at Different Times

**Figure 4:**
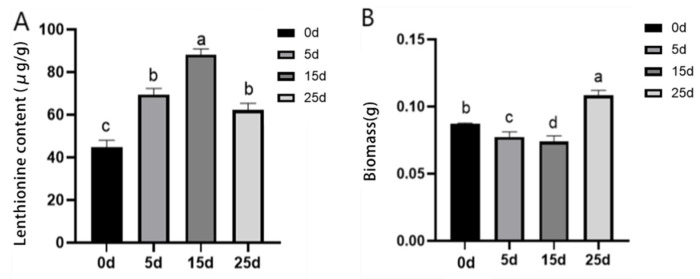
Effects of different durations of citric acid treatment on WT lenthionine content and biomass. (**A**) Effects of different durations of citric acid treatment on lenthionine content. (**B**) Effects of different durations of citric acid treatment on biomass. The calculation results are presented as mean ± standard deviation, and different letters indicate significant differences between different treatments (*p* < 0.05).

In order to investigate the effect of time on the induction of lenthionine following citric acid treatment, the lenthionine content, key enzyme activity, and biomass of two *L. edodes* species were examined by using different treatment durations. There was a significant difference in lenthionine when 5 days, 15 days, and 25 days were used as the treatment duration. At 15 days, *L. edodes* reached the peak level of lenthionine of 88.2 μg/g, which was 96% more than that of the control group (Figure 4A). Changes in biomass were examined in the citric acid treatment groups at different times. *L. edodes* reached its highest value at 25 days, where the maximum biomass of *L. edodes* was 0.11 g (Figure 4B). The above results indicate a significant response of *L. edodes* to the factor of treatment duration.

### 3.3. Response Surface Methodology to Optimize Citric Acid Treatment Conditions

The above experiments demonstrate that treatment with citric acid significantly affected the content of lenthionine without negative effects on biomass, both in terms of concentration and time. A five-level two-factor test was conducted using the central composite design (CCD) method in Design-Expert 12.0, with lenthionine as the target substance, and a total of 21 experimental groups were included, as shown in Table 1. The 21 combinations were re-run with different citric acid treatment durations and concentrations for fermentation tests to obtain the values of lenthionine content. The data were then analyzed using Design-Expert 12.0. The response surface model analysis is shown in Table 2, and the regression analysis is shown in Table 3. The quadratic multiple regression equations obtained after the software analysis were as follows:

Y = + 127.42 + 4.82 × Concentration + 6.97 × Time − 3.21 × Concentration × Time − 31.31 × Concentration^2^ − 28.17 × Time^2^

In the fitted equation, Y is the response value (the amount of lenthionine). The full-variance quadratic multiple regression model was analyzed, and the response surface resulting from the analysis was examined to obtain response surface contour plots, stereo plots, and normal probability plots of the standardized residuals. The regression analysis of the experimental results indicated that the effects of the primary terms of the equation, concentration and time, and the secondary terms, A^2^ and B^2^, were more significant. The F-value of the model is 25.48, implying that the model is significant and there is only a 0.01% chance that it may be affected by noise. The correlation coefficient R^2^ = 0.8947 indicates that 89.47% of the variation in lenthionine is distributed in the two factors of the equation and only 10.53% of the total variation in Y-values cannot be explained by the model, which implies a good fit between the actual and predicted values of lenthionine. The model Prob > F value was less than 0.0001, indicating that the fitted regression equation was suitable and a good fit for the experiment.

**Figure 5:**
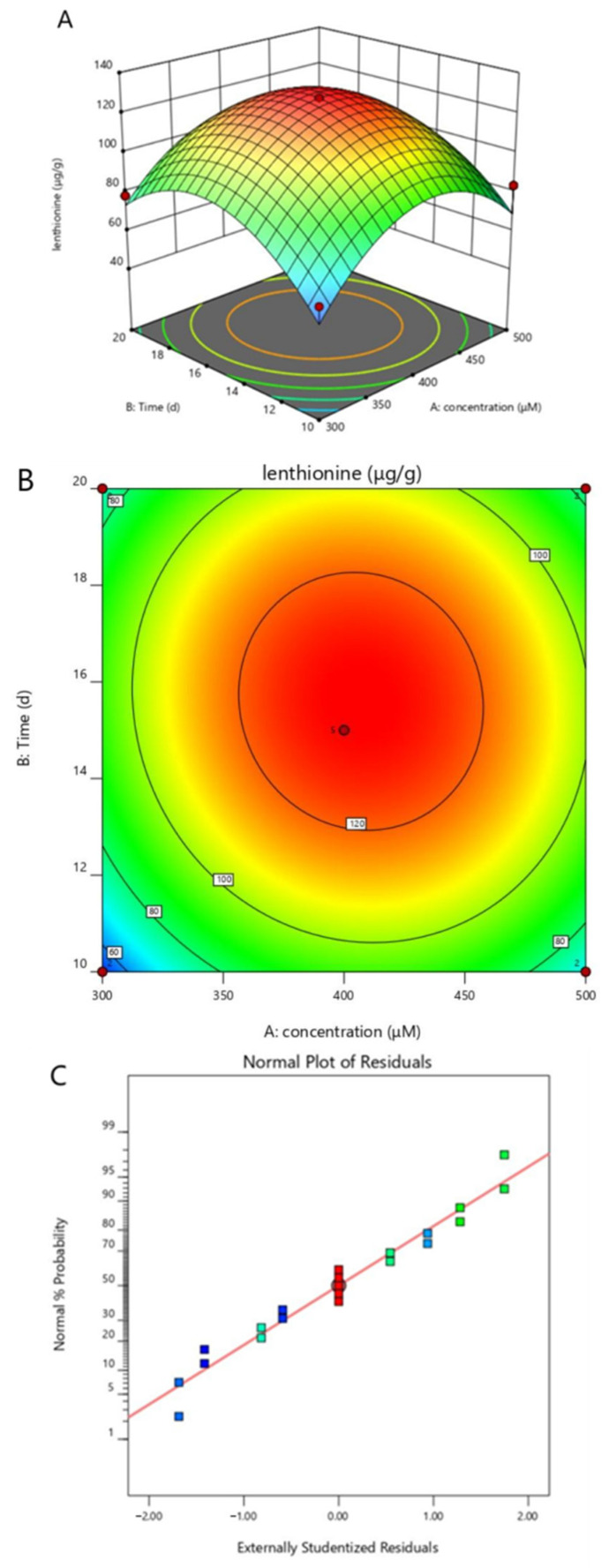
Optimization of citric acid treatment conditions for *L. edodes*. (**A**) A five-level two-factor central composite design is used to maximize the lenthionine of *L. edodes*. The independent variables are citric acid concentration and treatment duration. Table 1 gives the range and level of the independent variables, and the level of the variables is determined according to the univariate analysis. (**B**) The contour plot reflects the combined effects of concentration and treatment duration on the lenthionine of *L. edodes*. (**C**) Normal probability plot of normalized residuals.

*L. edodes* was affected by citric acid treatment concentration, treatment duration, and the interaction of these two factors. It can be seen that the normal probability distribution of the standardized residuals was concentrated relatively close to the straight line, with good data regression, indicating that the test model was reliable. The optimization results of the *L. edodes* citric acid treatment were finally obtained by combining the two factors to find the best results: the best citric acid treatment concentration was 406 μM and the best treatment duration was 15.6 d (Figure 5). The theoretical value of response value Y was 128 μg/g, which is close to the experimental replicate value of 130.84 μg/g. This indicates that it is feasible and reliable to optimize the treatment conditions for citric-acid-induced lenthionine in *L. edodes*, using a central combined experimental design.

### 3.4. Effect of Citric Acid on the Expression Levels of LEGGT and LECSL

**Figure 6:**
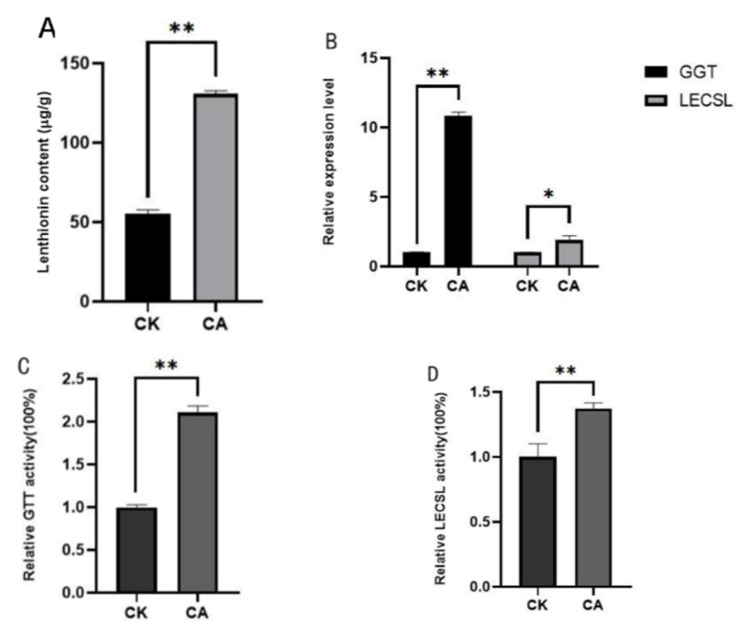
Lenthionine level, enzyme activity under citric acid treatment and the transcription level of the key synthesis gene. (**A**) Lenthionine content under 406 μM citric acid concentration. (**B**) The relative enzyme activities of LECSL and LEGTT. (**C**,**D**) qRT-PCR analysis of the expression of leggt and lecsl. The values are the mean ± standard deviations (SD) of the results of three independent experiments. The asterisks indicate significant differences compared with the untreated strains (Student’s *t*-test: * and ** *p* < 0.01).

The *L. edodes* was treated with the optimal conditions of citric acid treatment obtained above and the examined gene expression levels of LEGGT and LECSL. The content of lenthionine appeared to be increased under citric acid treatment, as expected (Figure 6A). A 2.1-fold increase in the enzyme activity level of LEGGT and a 1.3-fold increase in the enzyme activity of LECSL was observed based on the assays of LEGGT and LECSL (Figure 6C,D). To investigate the expression levels in *L. edodes* under citric acid induction conditions, the transcript levels of LEGGT and LECSL were examined following citric acid treatment. The genes coding for LEGGT and LECSL showed a higher transcript level under citric acid treatment, compared with the control group (Figure 6B). The above experimental results show that citric acid treatment significantly activated the transcript levels of LEGGT and LECSL, and increased the enzymatic activity level of both LEGGT and LECSL.

### 3.5. LEGGT and LECSL Genes Play an Important Role in the Citric-Acid-Induced Biosynthesis of Lenthionine

**Figure 7:**
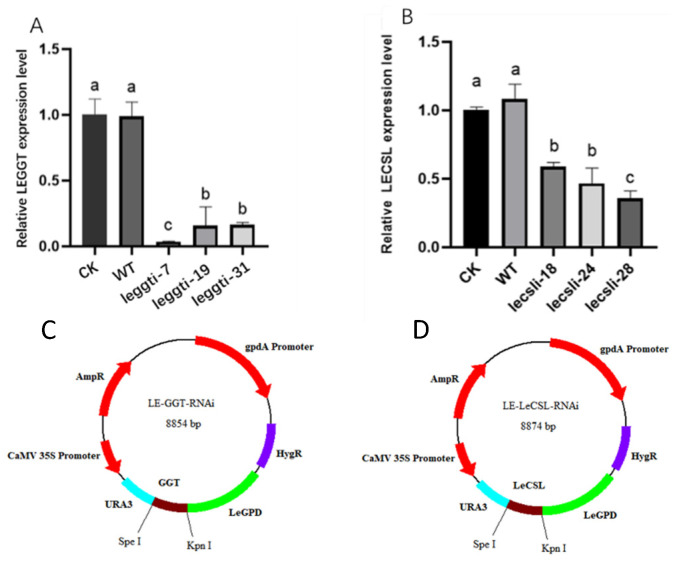
Construction and screening of *L. edodes* LEGGT- and LECSL-silenced strains. (**A**) Detection of LEGGT gene transcript levels in LEGGT-silenced strains. (**B**) Detection of LECSL gene transcript levels in LECSL-silenced strains. The values in each column represent the fluorescence ratio in each captured image and are shown as the mean ± SD. The asterisks indicate significant differences compared with the untreated strains (Student’s *t*-test: *p* < 0.01). (**C**) Construction of the LEGTT RNAi expression cassette plasmids. The LEGTT fragment was double-digested with the restriction enzymes KpnI and SpeI, then inserted into the pAN7-dual plasmid. In the plasmid, ura3 transcription is driven by the 35S and gpd promoters. (**D**) Construction of the LECSL RNAi expression cassette plasmids. The calculation results are presented as mean ± standard deviation, and different letters indicate significant differences between different treatments (*p* < 0.05).

To clarify the mechanism of action of LEGTT and LECSL under citric acid induction, the silencing plasmid LE-GTT-RNAi of GTT was constructed using RNAi technology. Three transformants (legtti-7, leggti-19, and leggti-31), with a silencing efficiency of 70%, were screened using fluorescent quantitative PCR to analyze LEGGT gene expression (Figure 7A). The LECSL silence transformants (lecsli-18, lecsli-24, and lecsli-28) were constructed in the same way (Figure 7B).

**Figure 8:**
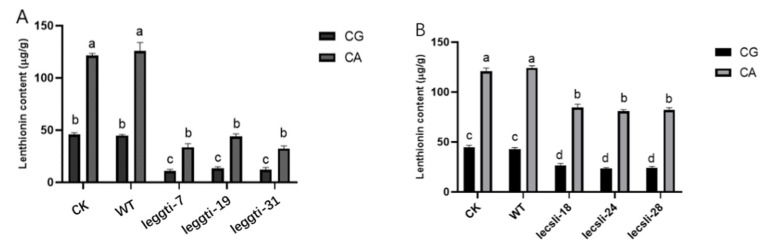
Variation in lenthionine levels and enzyme activity in silenced strains under citric acid treatment. (**A**) Lenthionine levels of LEGGT-silenced strains under citric acid treatment (CG: control group; CA: citric acid treatment). (**B**) Lenthionine levels of LECSL-silenced strains under citric acid treatment. The calculation results are presented as mean ± standard deviation, and different letters indicate significant differences between different treatments (*p* < 0.05).

To study the importance of LEGGT expression in the regulation of lenthionine by citric acid, the lenthionine content of CK and WT strains and LEGGT-silenced strains (leggti-7, leggti-19, and leggti-31) were measured under citric acid treatment. The lenthionine of the LEGGT-silenced strains leggti-7, leggti-19, and leggti-31 decreased significantly under untreated conditions. The lenthionine content of the LEGGT-silenced strains leggti-7, leggti-19, and leggti-31 also increased under citric acid treatment, although the increase did not reach the same level as that of WT and CK (Figure 8A). To investigate the effect of LECSL on lenthionine, the lenthionine content of CK (no-load control) and WT strains and LECSL-silenced strains (lecslti-18, lecsli-24, and lecsli-28) were examined. There was a significant decrease in the content of lenthionine in the LECSL-silenced strains, probably due to the low silencing efficiency of the strains, which showed a lower decreasing trend than the LEGGT-silenced strains. Although the lenthionine of the citric-acid-treated silenced strain was lower than that of the CK and WT strains in the same treatment group, the lenthionine was still higher than that of the non-citric-acid-treated strain (Figure 8B).

The above results show that change” in ’EGGT and LECSL were ©mportant factors for the changes in lenthionine under citric acid treatment conditions.

## 4. Discussion

The aroma component is an important commercial feature of edible fungi, which is the reason for the popularity of edible fungi among consumers. The essence of *Lentinula edodes* is the basis of its unique aroma and is one of the important indicators of odor. Therefore, it is of great significance to study the synthesis mechanism of the aroma components of *Lentinula edodes* in order to improve its commercial value.

The addition of inducers or increasing the composition of the culture medium are novel methods of improving biosynthesis. The research of Hiraide et al. shows that the content of lenthionine increases with the addition of Cys (300 mg/kg) and methionine (400 mg/kg). If Glu (17 g/kg) is added, the content of odor compounds will further increase [26]. These amino acids exist as substrates for the synthesis of lentinic acid, which leads to the increase in the content of lenthionine. In this study, *L. edodes* was treated with citric acid of a lower concentration (56.6 mg/kg), and the content of lenthionine was increased. Therefore, this study speculated that citric acid was not used as a substrate but as an inducer to participate in the biosynthesis of lenthionine. There are a few studies on citric acid as an inducer to regulate fungal growth and development, and the content of secondary metabolites, especially in *L. edodes*. This paper found that exogenous citric acid treatment can significantly improve the content of lenthionine, and this increase is affected by the dose and duration of treatment. The best citric acid concentration for Lentinula edodes was 300 μM, and the best treatment duration was 15 days. Based on these values, the optimal induction concentration and duration were obtained through surface response (RSM) optimization, which provides a basis for further revealing the mechanism of how citric acid improves the content of shiitake essence. The possible action sites of citric acid in the biosynthesis of lentinan were preliminarily discussed, providing a reference for the effective application of citric acid as an inducer in fungi.

In the process of lenthionine synthesis, the key enzyme γ-glutamyl transpeptidase (LEGGT) hydrolyzes the glutamine peptide bond in lentinic acid to produce glutamyl lentinic acid and glutamic acid as a by-product. Cystine sulfoxide lyase (LECSL) acts to further convert the product into thiosulfinate, which is an unstable and highly reactive intermediate [9]. There are similar metabolic pathways for the polysulfide flavor compound allicin, found in garlic. Therefore, LEGGT and LECSL are analogized to the key enzymes in the allicin biosynthesis pathway, γ-glutamyl transpeptidase, and alliinase. A key enzyme in the biosynthetic pathway, γ-glutamyl transpeptidase (AsGGT1, AsGGT2, and AsGGT3), regulates the content of alliin (S-allyl-L-cysteine), the precursor substance of allicin, which, in turn, is produced by alliinase. The yield of allicin is closely related to the regulation of alliinase activity [7,27]. Although these compounds are very different from the final products of *Lentinula edodes* in terms of chemical structure and reactivity, *L. edodes* and sulfurous allium end products are all derived from a family of γ-L-glutamyl-cysteine sulfoxide precursors [28]. Wang et al. found that polyhydroxy compounds, such as glucose, sucrose and glycerin, have certain activation effects on alliinase, and these inducers increase allicin content through this metabolic pathway [7]. Therefore, we speculate that citric acid, as an inducer, can enhance LEGGT and LECSL activity to regulate the biosynthesis of lenthionine. In order to investigate why citric acid affects lenthionine at the genetic level, we examined the expression levels of LEGGT- and LECSL-coding genes in *L. edodes* under citric acid treatment, and found that the expression levels of both LEGGT- and LECSL-coding genes showed a significant increase, with LEGGT being more significantly affected. Furthermore, the silenced strains of LEGGT and LECSL were constructed separately using RNAi, and the lenthionine of the silenced strains was detected under citric acid treatment. The overall lenthionine levels of both silenced strains decreased greatly, but showed a small increase under citric acid treatment, although this increase was much lower than that of the WT and CK strains.

## 5. Conclusions

In conclusion, this experiment proved that citric acid can induce the accumulation of lenthionine in *L. edodes*. In this paper, the single-factor treatment of *Lentinula edodes* with variable citric acid concentration and treatment duration showed that the best citric acid treatment concentration for Lentinula edodes was 300 μM/L, and the best treatment duration was 15 days. Additionally, the optimal design conditions were obtained using the response surface method (RSM): the treatment concentration was 406 μM/L, the treatment duration was 15.6 days, and the lenthionine content was 130 μg/g. The expression levels of LEGGT and LECSL genes increased significantly under citric acid treatment. The lenthionine content of the LEGGT- and LECSL-silenced strains was significantly decreased. The results indicate that LECSL and LEGGT are involved in the regulation of the biosynthesis of lenthionine, induced by citric acid in *L. edodes.* If the low-cost application of a citric acid inducer can effectively improve the quality and cultivation efficiency of Lentinula edodes, this will result in great progress in the development of the Lentinula edodes industry.

## Figures and Tables

**Table 1:** Design matrix of *L. edodes* citric acid CCD.

Run	A: Concentration (μM)	B: Time (d)	Lenthionine (μg/g)
1	400	15	127.42
2	400	15	127.42
3	258.579	15	52.5612
4	300	20	78.2731
5	400	15	127.42
6	541.421	15	57.3184
7	541.421	15	57.3184
8	400	15	127.42
9	400	7.92893	48.9913
10	400	22.0711	73.5658
11	500	20	87.6737
12	500	10	83.6007
13	300	10	61.356
14	400	7.92893	48.9913
15	500	20	87.6737
16	300	10	61.356
17	258.579	15	52.5612
18	400	15	127.42
19	500	10	83.6007
20	400	22.0711	73.5658
21	300	20	78.2731

Note: The central composite design includes a total of 21 experimental combinations. Each experiment was repeated three times and the mean lenthionine value was calculated. The experimental data were analyzed by a second-order polynomial regression.

**Table 2:** ANOVA for response surface quadratic model.

Source	Sum of Squares	DF	Mean squares	F Value	*p*-Value (Prob > F)
Model	14,795.45	5	2959.09	25.48	<0.0001
Residual	1741.84	15	116.12		
Lack of fit	1741.84	3	580.61		
Pure error	0.0000	12	0.0000		
Cor total	16,537.29	20			

**Table 3:** The least-squares fit and coefficient estimate.

Factor	Coefficient Estimate	df	Standard Error	95% CI Low	95% CI High	VIF
Intercept	127.42	1	4.82	117.15	137.69	
A: time	4.80	1	2.69	−0.9455	10.54	1.0000
B: concentration	6.97	1	2.69	1.23	12.71	1.0000
AB	−3.21	1	3.81	−11.33	4.91	1.0000
A^2^	−31.34	1	3.35	−38.48	-24.19	1.14
B^2^	−28.17	1	3.35	−35.32	-21.02	1.14

## Data Availability

Data is contained within the article or Supplementary Materials.

## Supplementary Materials

The following supporting information can be downloaded at: https://www.mdpi.com/article/10.3390/foods11244110/s1, Table S1. Primers used in the article.
