# Supplementary material for: Citric Acid Induces the Increase in Lenthionine Content in Shiitake Mushroom, Lentinula edodes
Source: Foods. 2022 Dec 19;11(24):4110. doi: 10.3390/foods11244110 (PMC9777562; doi:10.3390/foods11244110)
Supplement: Supplementary file 1 [file foods-11-04110-s001.zip › foods-2024324-supplementary.pdf]

Table S1. Primers used in the article

Primer	Sequence (5′to 3′)	Description
RT-Actinl-F	GGAGAAGATTTGGCATCACACA	Detects the Actinl expression
RT-Actinl-R	GAAGAGCGAAACCCTCGTAGA	
RT- LEGGT-F	CGCAACCCCGCTTATCT	Detects the *LEGGT* expression
RT- LEGGT-R	CGTCAACCGCTGAACCA	
RT-LECSL-F	CTTCTTTTCCCCACCACC	Detects the *LECSL* expression
RT-LECSL-R	TCCTTCCACGGCATAGC	
